# Significance of PI3K/AKT signaling pathway in metastasis of esophageal squamous cell carcinoma and its potential as a target for anti-metastasis therapy

**DOI:** 10.18632/oncotarget.16333

**Published:** 2017-03-17

**Authors:** Bin Li, Wen Wen Xu, Alfred King Y. Lam, Yang Wang, Hui-Fang Hu, Xin Yuan Guan, Yan Ru Qin, Nassim Saremi, Sai Wah Tsao, Qing-Yu He, Annie L.M. Cheung

**Affiliations:** ^1^ School of Biomedical Sciences, Li Ka Shing Faculty of Medicine, The University of Hong Kong, Pokfulam, Hong Kong SAR, China; ^2^ College of Life Science and Technology, Jinan University, Guangzhou, China; ^3^ Centre for Cancer Research, Li Ka Shing Faculty of Medicine, The University of Hong Kong, Pokfulam, Hong Kong SAR, China; ^4^ The University of Hong Kong-Shenzhen Institute of Research and Innovation (HKU-SIRI), Shenzhen, China; ^5^ Department of Pathology, Griffith Medical School and Menzies Health Institute Queensland, Gold Coast Campus, Gold Coast, QLD, Australia; ^6^ Department of Clinical Oncology, Li Ka Shing Faculty of Medicine, The University of Hong Kong, Pokfulam, Hong Kong SAR, China; ^7^ Department of Clinical Oncology, First Affiliated Hospital, Zhengzhou University, Zhengzhou, China

**Keywords:** PI3K/AKT, esophageal squamous cell carcinoma, metastasis, targeted therapy

## Abstract

Metastasis is the most lethal hallmark of esophageal squamous cell carcinoma (ESCC). The aim of the study is to identify key signaling pathways that control metastasis in ESCC. Highly invasive ESCC sublines (designated I3 cells) were established through three rounds of selection of cancer cells invading through matrigel-coated chambers. Gene expression profile of one of the I3 sublines was compared with that of its parental cell line using cDNA microarray analysis. Gene ontology and pathway analyses of the differentially expressed genes (both upregulated and downregulated) indicated that genes associated with cellular movement and the AKT pathway were associated with increased cancer cell invasiveness. Western blot analysis confirmed increased phosphorylated AKT (p-AKT), N-cadherin and decreased E-cadherin expression in the I3 cells. Immunohistochemistry was used to evaluate the clinical significance of p-AKT expression in ESCC, and the results showed higher p-AKT nuclear expression in lymph node metastases when compared with primary carcinoma. Inactivation of the PI3K/AKT pathway with specific inhibitors, or with PTEN overexpression, resulted in reversed cadherin switching and inhibited cancer cell motility. Inhibition of the pathway by treatment with wortmannin markedly suppressed experimental metastasis in nude mice. Our data demonstrated the importance of the PI3K/AKT signaling pathway in ESCC metastasis and support PI3K/AKT as a valid therapeutic target in treatment of metastatic ESCC.

## INTRODUCTION

Esophageal cancer is associated with poor prognosis because over 50% of patients with esophageal cancer present with metastasis [1]. Cancer metastasis is a multi-stage process which involves enhanced cell motility, intravasation, transit in lymphatic and blood vessels, extravasation and finally growth at a new location. Each of these events is attributed, at least in part, to the acquisition of genetic alterations in the tumor cells [2]. Identification of critical regulators and signaling pathways that drive invasion and metastasis will facilitate development of new treatment strategies.

In the present study, we established highly invasive ESCC sublines (designated I3 cells) by serial selection of cancer cells invading through matrigel-coated Boyden chambers to explore the mechanisms involved in metastasis in ESCC. Bioinformatics analysis of gene expression profile of the I3 cells, followed by confirmation of increased phosphorylated-AKT (p-AKT) expression in I3 cells and in metastatic ESCC in lymph nodes, suggested that the phosphatidylinositol-3-kinase (PI3K)/AKT pathway has a crucial role in esophageal cancer invasion and metastasis. The PI3K/AKT signaling pathway is implicated in a variety of oncogenic processes including cell proliferation, survival, epithelial mesenchymal transition (EMT), enhanced motility, and angiogenesis [3–6]. Our previous study demonstrated that pharmacological inhibition of the PI3K/AKT pathway can offset the increased invasiveness and motility induced by Id1-overexpression in ESCC cells [7]. However, the effects of PI3K inhibitors on suppressing the metastatic potential of non-transfected ESCC cells have not been reported previously and warrant further investigation. LY294002 and wortmannin are potent PI3K inhibitors that are commonly used in laboratory and preclinical studies but have been precluded from clinical use due to instability, poor solubility and/or high toxicity. Since new evidence supports that the clinical application of wortmannin may be revived through nanoparticle drug delivery [8], wortmannin was used in this study to investigate the effects of PI3K/AKT blockade on *in vivo* metastasis of human ESCC cells in mice. Moreover, because increased invasiveness may be conferred by EMT during which epithelial markers are usually downregulated while mesenchymal markers are upregulated, we also examined the expression levels of EMT markers including E-cadherin and N-cadherin in ESCC cells (including the I3 cells), and determined whether PI3K/AKT inhibition by LY294002 and wortmannin could reverse the EMT program.

## RESULTS

### KYSE410-I3 and KYSE510-I3 sublines are highly invasive and show increased EMT

**Figure 1:**
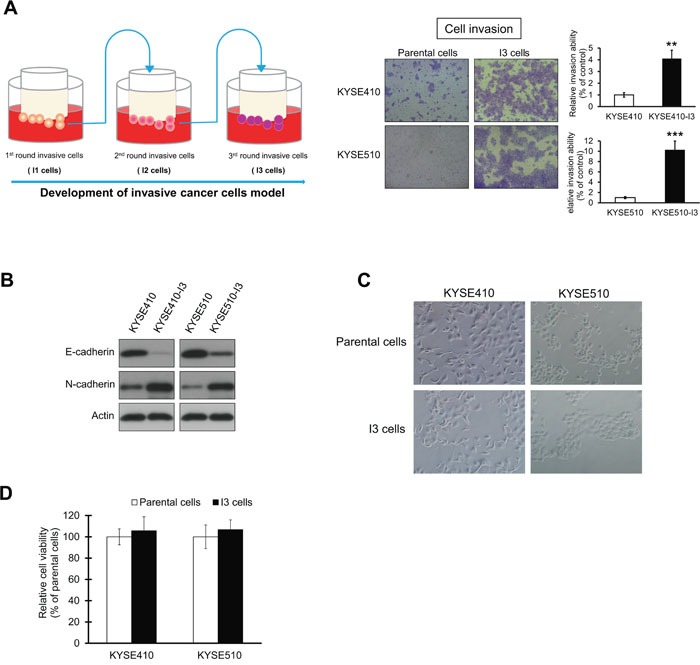
Establishment of highly invasive ESCC sublines **(A)** Matrigel chamber invasion assay comparing the invasive potential of KYSE410-I3 and KYSE410-I3 sublines with that of corresponding parental cells. The quantification data show dramatic increase in invasive potential of I3 cells. **(B)** Comparison of E-cadherin and N-cadherin expressions in I3 cells and parental cells. **(C)** Morphology of I3 cells and parental cells. **(D)** Parental and I3 cells had similar proliferation rates as determined by MTT assay. Bars, SD; **, *P* < 0.01; ***, *P* < 0.001 compared with control cells.

The KYSE410-I3 and KYSE510-I3 sublines showed significantly higher invasive potential (Figure 1A), and enhanced EMT as indicated by marked decrease in E-cadherin and increase in N-cadherin expression (Figure 1B), compared with their respective parental ESCC cell lines, although no significant difference in morphology was observed (Figure 1C). The comparable proliferation rates of the I3 cells and parental cells within a 24-hour time frame ruled out the possibility that the increase in evaded I3 cells in the cell invasion assay was due to increased proliferation (Figure 1D).

### Highly invasive esophageal cancer cells overexpress p-AKT

**Figure 2:**
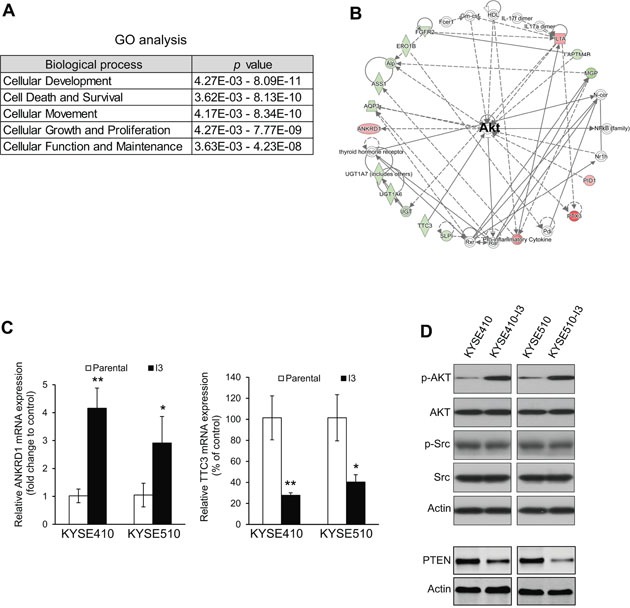
Highly invasive ESCC cells exhibit PI3K/AKT activation **(A)** GO analysis of cDNA microarray data from KYSE410-I3 cells and KYSE410 cells. **(B)** Ingenuity Pathway Analysis (IPA)indicated a dysregulation of AKT signaling in I3 cells. **(C)** Comparison of mRNA expression levels of *ANKRD6* and *TTC3* in I3 cells and corresponding parental cells by qRT-PCR. **(D)** Western blot analysis of expression levels of p-AKT, AKT, PTEN, p-Src and Src in I3 sublines and corresponding parental cells.

The gene expression profiles of KYSE410-I3 and its parental cell line were compared using cDNA microarray. Of the 246 differentially expressed genes in KYSE410-I3, 232 (including 63 upregulated and 169 downregulated genes (listed in Supplementary Table 1) were mapped to known functions and pathways by IPA. Gene Ontology (GO) analysis indicated that the differentially expressed genes in the I3 cells were significantly associated with five important cellular functions including cell movement (Figure 2A). Pathway analysis showed that a cluster of differentially expressed genes in the I3 cells constitute a signaling network with AKT as central hub (Figure 2B), thus suggesting dysregulation of AKT signaling in these cells. The upregulation and downregulation of representative genes including *ANKRD1* and *TTC3*, which were randomly selected from the list of differentially expressed genes, were confirmed by quantitative RT-PCR (Figure 2C). Western blot analysis confirmed upregulation of p-AKT expression, and significantly reduced expression level of PTEN which is the upstream negative regulator of PI3K/AKT pathway, in KYSE410-I3 and KYSE510-I3 cells (Figure 2D). Interestingly, there was no activation of the Src pathway, which is another important regulatory pathway in cancer cell invasion and metastasis [9, 10], as indicated by the comparable protein expression level of p-Src and Src between the I3 sublines and corresponding parental cells (Figure 2D), even though the IPA analysis also suggested the dysregulation of the Src pathway in KYSE410-I3 cells. This further highlighted the significance of PI3K activation in esophageal cancer invasion and metastasis.

### Inhibition of PI3K/AKT signaling reduces esophageal cancer cell invasion and migration

**Figure 3:**
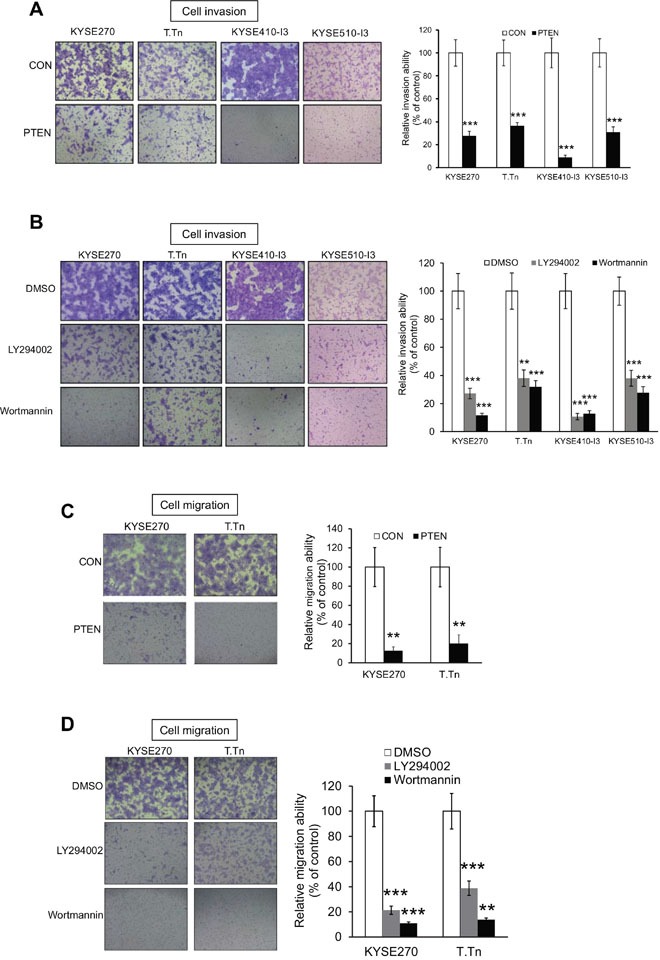
Inhibition of PI3K/AKT pathway suppresses esophageal cancer cell invasion and migration **(A)** Human esophageal cancer cells KYSE270, T.Tn, KYSE410-I3 and KYSE510-I3 with PTEN overexpression were subjected to invasion assay. Values were then normalized to cells expressing vector control (CON). **(B)** Treatment with 5 μM LY294002 or 5 μM wortmannin reduced the invasive potential of KYSE270, T.Tn, KYSE410-I3, and KYSE510-I3 cells as determined by chamber invasion assay. **(C)** PTEN overexpression inhibited cell migration in KYSE270 and T.Tn cells as determined by chamber migration assay. **(D)** PI3K/AKT inhibitors, LY294002 and Wortmannin, significantly suppressed cancer cell migration in KYSE270 and T.Tn cells. Bars, SD; **, *P* < 0.01; ***, *P* < 0.001 compared with control cells.

To study whether PI3K/AKT inhibition can suppress esophageal cancer cell motility and reverse the invasiveness of I3 cells, a vector expressing *PTEN* was transfected into KYSE410-I3 and KYSE510-I3 cells, as well as KYSE270 and T.Tn which were ESCC cell lines with relatively high invasive ability. Our results showed that PTEN overexpression significantly reduced the ability of esophageal cancer cells to invade (Figure 3A). Treatment with a low concentration (5 μM) of LY294002 or wortmannin, which had no significant inhibitory effects on cell proliferation of these cells within 24 hours [11], also markedly inhibited ESCC cell invasion (Figure 3B). Likewise, cell migration assays showed that inhibition of PI3K/AKT signaling by overexpressing *PTEN* (Figure 3C) or pharmacological blockade (Figure 3D) markedly retarded ESCC cell migration.

### Blockade of PI3K/AKT reverses cadherin switching

**Figure 4:**
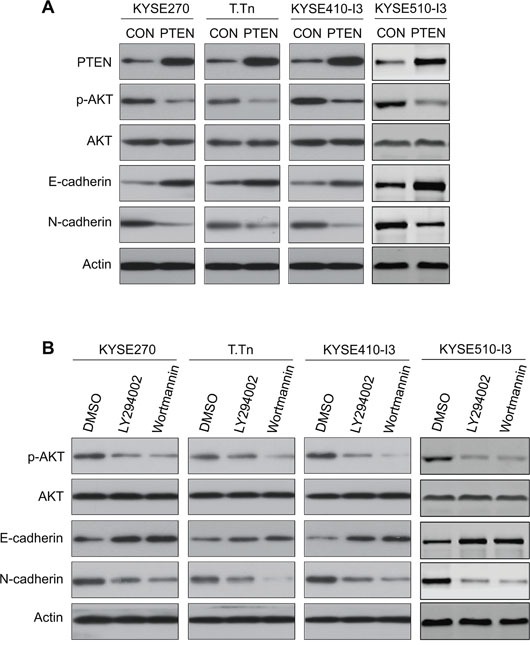
Effects of PI3K/AKT blockade on E-cadherin and N-cadherin expressions in esophageal cancer cells **(A)** Western blot analysis showed that PTEN overexpression in KYSE270, T.Tn, KYSE410-I3, and KYSE510-I3 cells increased the expression level of E-cadherin and decreased N-cadherin. Actin was included as internal loading control. **(B)** Western blot analysis of protein expressions of E-cadherin and N-cadherin in esophageal cancer cells treated with wortmannin or LY294002 for 24 h compared with the DMSO-treated cells.

Cadherin switching, which is characterized by downregulation of E-cadherin and upregulation of N-cadherin expression, is considered a hallmark of EMT and cancer invasion [12]. To study whether the effects of PI3K/AKT inhibition on esophageal cancer cell motility were associated with changes in E-cadherin and N-cadherin expressions, the esophageal cancer cells transfected with *PTEN* or treated with PI3K/AKT inhibitors were analyzed by Western blot. The increase in E-cadherin and decrease in N-cadherin expression levels upon PI3K/AKT inhibition suggests that the effects of PI3K/AKT blockade on esophageal cancer cell mobility were due to reversal of EMT (Figure 4A and 4B).

### Wortmannin suppresses metastasis of esophageal cancer cells in nude mice

**Figure 5:**
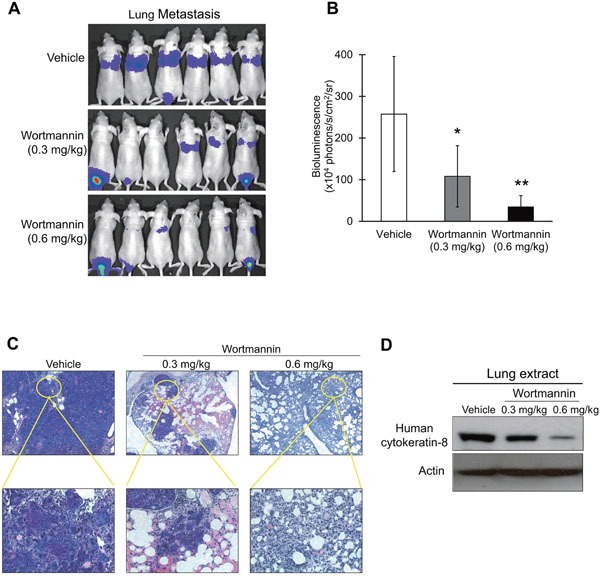
Effect of wortmannin treatment on metastasis of ESCC cells in nude mice **(A-C)** Human esophageal cancer cells with ectopic luciferase (Luc) expression, KYSE150-Luc, were intravenously injected into nude mice through the tail vein. The mice were treated with 0.3 mg/kg or 0.6 mg/kg wortmannin, or DMSO twice a week. **(A)** Bioluminescent imaging of luciferase-expressing esophageal cancer cells eight weeks later. **(B)** Quantification of bioluminescent signals by Living Image software. Note that treatment with wortmannin significantly reduced the metastatic activity of esophageal cancer cells in a dose-dependent manner. **(C)** Micrometastases in the lungs were detected histologically. **(D)** The lung extracts from wortmannin-treated mice were compared with those of DMSO-treated mice for human-specific cytokeratin-8 expression level by western blot. Bars, SD; *, *P* < 0.05; **, *P* < 0.01 compared with DMSO-treated mice.

Since our results showed that PI3K/AKT inhibition reduced the migration and invasive potential of esophageal cancer cells *in vitro* (Figure 3), we evaluated the efficacy of PI3K/AKT blockade in inhibiting metastasis of esophageal cancer cells *in vivo*. Nude mice were intravenously inoculated with luciferase-expressing esophageal cancer cells, and treated with different doses of wortmannin. Bioluminescent imaging and histological examination of the lungs demonstrated that wortmannin treatment significantly suppressed the metastatic colonization of esophageal cancer cells to the lungs in a dose-dependent manner (Figure 5A, 5B and 5C). Moreover, the suppressive effect of wortmannin on metastasis was confirmed by the lower expression of human-specific cytokeratin-8 (expressed by human ESCC cells) in the lung extracts of wortmannin-treated mice compared with the control group (Figure 5D). The presence of bioluminescent signals in the tail region of some of the animals in the wortmannin treatment groups could be due to proliferation of small number of injected cancer cells which had leaked into the surrounding tissue during or after tail vein injection, but were subsequently inhibited from entering the circulation and undergoing metastatic seeding by the anti-invasive and anti-migratory effects of wortmannin.

### PI3K/AKT pathway is constitutively activated in lymph node metastases of ESCC

**Figure 6:**
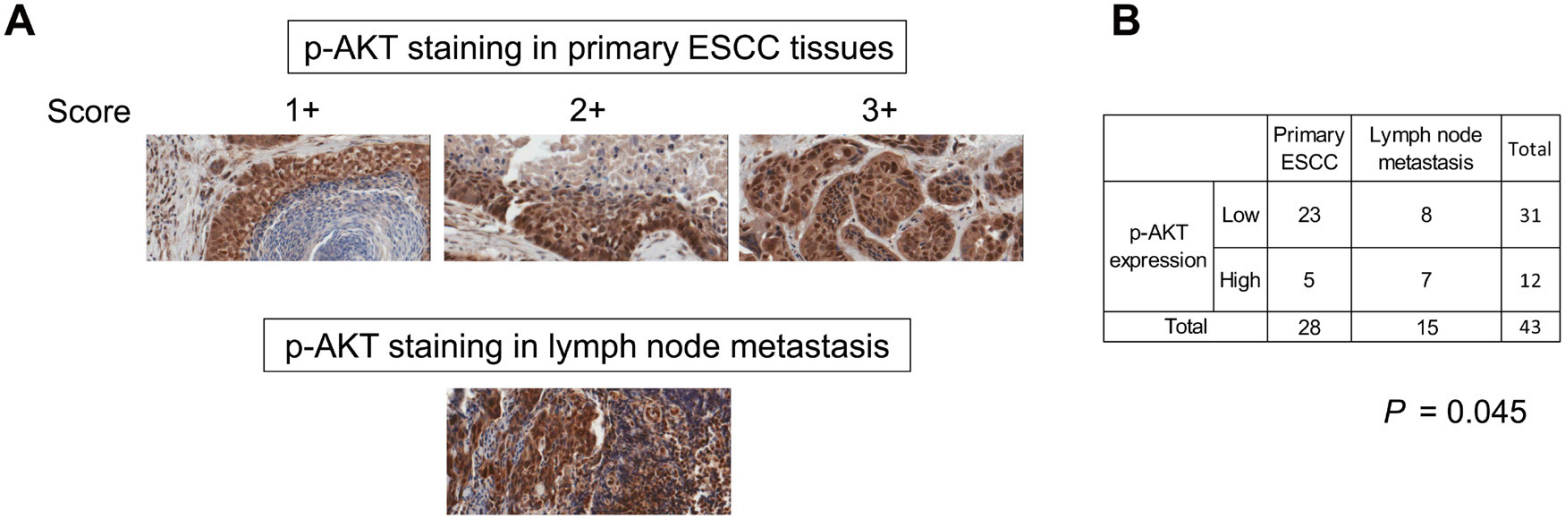
Constitutive activation of PI3K/AKT in lymph node metastases of ESCC **(A)** Representative immunohistochemical images of p-AKT expression (x 20 magnification) in primary ESCC and lymph node metastases. Note that the lymphocytes were also positively-stained. **(B)** Pearson's Chi-Square test showed significantly higher p-AKT expression in metastatic carcinoma compared with primary tumors.

To determine whether PI3K/AKT activation is clinically relevant in the pathogenesis of lymph node metastasis in ESCC, the expression level of p-AKT in ESCC tissue was determined immunohistochemically. Positive staining was mainly detected in the nuclei of cancer cells in the primary carcinoma and in lymph node metastases (Figure 6A). Among 28 cases of primary tumors, only 5 cases had high p-AKT nuclear expression, compared to 7 out of 15 cases of lymph node metastases (*P* = 0.045), indicating that there was a higher percentage of ESCC cells with activated AKT in the lymph node metastases than in the primary tumors (Figure 6B). These data highlighted the significance of PI3K/AKT signaling pathway in metastasis in ESCC and its potential as therapeutic target in cancer treatment. However, analysis of p-AKT expression in a tissue microarray (TMA) containing primary ESCC tissues did not yield any significant correlation with TNM pathological staging (Supplementary Table 2).

## DISCUSSION

Cancer metastases, rather than the primary cancer, account for as high as 90% of cancer death [13, 14]. Recent advances in gene profiling techniques and establishment of metastatic cell models have been instrumental in identifying genes and pathways associated with tumor metastasis [15]. To our knowledge, this is the first study that compares the gene profiles of highly invasive ESCC cells and the relatively non-invasive parental cells. The highly invasive ESCC sublines that were established in this study not only confirms the significance of the PI3K/AKT pathway in ESCC metastasis, but will be valuable tools for evaluating novel therapeutic agents which can curb tumor metastasis.

Although the PI3K/AKT signaling pathway is well documented to drive EMT, invasion and metastasis in human cancer [16–20], there are surprisingly few reports on its physiological significance in metastasis of ESCC. The metastasis-promoting function of Id1, MMP-1, integrin alpha6 and PITX2, for example, requires activation of the PI3K/AKT pathway [7, 21–23]. Here, our results showed that p-AKT expression was increased in the highly invasive esophageal cancer sublines and was likely due to downregulation of PTEN. Loss of E-cadherin, the key gatekeeper of the epithelial state, is necessary for the EMT process, and has been associated with cancer progression and poor prognosis in ESCC [24, 25]. N-cadherin, a mesenchymal cadherin associated with EMT, promotes tumor cell invasion and metastasis, and its high expression is associated with metastasis in ESCC [26–28]. Notably, E-cadherin to N-cadherin switching is necessary for increased motility though not required for morphological changes that accompany EMT [29]. We found that pharmacological and genetic blockade of PI3K/AKT pathway significantly inhibited migration and invasion in esophageal cancer cells, accompanied by upregulation of E-cadherin and downregulation of N-cadherin. The mechanisms by which activation of the PI3K/AKT pathway induces cadherin switching warrant further investigation, but previous studies have provided some clues. It was reported that E-cadherin is repressed by NFκB, the downstream pathway of PI3K/AKT signaling, and that this effect is mediated by multiple transcriptional regulators of E-cadherin including the Snail family of transcriptional repressors, Snail and Slug [30], and the ZEB family members, ZEB1 and ZEB2 [31]. In addition, PI3K/AKT can upregulate mRNA expression of N-cadherin through transcriptional regulation of Twist and Snail [32, 33].

Several lines of evidence indicate that translocation of activated/phosphorylated AKT to the nucleus is important for regulation of signaling pathways involved in proliferation and survival of cancer cells [34]. Our immunohistochemical evaluation of primary ESCC showed no significant correlation between p-AKT nuclear expression and pathological stage, which was consistent with what was described in two recent studies [35, 36] reporting that low p-AKT alone or concurrent high EGFR and low p-AKT expression is associated with better outcome in esophageal cancer patients who received chemotherapy, but p-AKT alone may not be an ideal predictor of survival for patients without chemotherapy. Although our results showed a lack of correlation between p-AKT expression and M-stage, which could be due to the small number of cases with distant metastasis (n = 2) in our sample cohort, a significantly higher p-AKT expression was found in metastatic ESCC in lymph nodes, suggesting that PI3K/AKT activation is critical for metastatic colonization which is one of the most ominous features of cancer progression [37]. Since cancer cells are frequently present in the blood and distant organs of cancer patients at initial presentation, it has been suggested that the capability of therapeutic agents to impair proliferation of already disseminated cancer cells should be a key consideration in drug design [2]. Unfortunately, many of the current compounds in clinical trials and preclinical studies such as matrix metalloproteinases inhibitors and Axl kinase inhibitor act principally by blocking the escape of cancer cells from primary tumors [38]. The experimental metastasis model used in the present study in which intravenously injected cancer cells colonized distant organs to form micro- and macro-metastases best mimics the most crucial and selective steps of metastasis. We have provided direct evidence that treatment with PI3K/AKT inhibitor can significantly suppress metastasis of ESCC cells in an *in vivo* setting.

Emerging evidence suggests that tumor microenvironment and angiogenesis also play critical roles in metastasis [39–41]. A recent study showed that the binding of tumor-associated macrophages to cancer cells through vascular cell adhesion molecule 1 (VCAM1) activates AKT signaling and protects cancer cells from apoptosis in the lung microenvironment [42]. Collectively, these evidences support the rationale for PI3K/AKT blockade to suppress micrometastasis formation and metastatic colonization. Further studies on the efficacy of PI3K/AKT blockade in preventing the earlier steps of metastasis in ESCC will enhance our understanding of the role of PI3K/AKT pathway in this process. However, unlike breast [43], lung [44], and liver cancers [45] in which spontaneous metastasis mouse models are commonly used to mimic all the steps of tumor metastasis, such models are not readily available for ESCC. In summary, our results confirm the importance of the PI3K/AKT pathway in metastasis and support its potential applicability as a therapeutic target in ESCC.

## MATERIALS AND METHODS

### Cell culture and drugs

Human ESCC cell lines KYSE150, KYSE270, KYSE410 and KYSE510 obtained from DSMZ (Braunschweig, Germany) [46], and T.Tn obtained from Dr. Hitoshi Kawamata (Dokkyo University School of Medicine, Tochigi, Japan) [47], were maintained in RPMI 1640 (Sigma, St. Louis, MO, USA) supplemented with 10% fetal bovine serum (Invitrogen, Gaithersburg, MD, USA) at 37°C in 5% CO_2_. Wortmannin and LY294002 were purchased from Calbiochem (Darmstadt, Germany) and dissolved in dimethyl sulfoxide (DMSO).

### Establishment of highly invasive esophageal cancer sublines

Esophageal squamous cell carcinoma cell lines KYSE410 and KYSE510, which had relatively weak invasive ability, were seeded into the upper compartment (4 × 10^5^ cells/well) of an 8 μm pore size BioCoat Matrigel Invasion chamber (BD Biosciences, Bedford, MA, USA) and incubated for 48 h. The cells that invaded and adhered on the lower surface of chamber were detached with trypsin and cultured until the number of cells was adequate for the next round of invasion selection, then the cells were re-inoculated into the upper compartment of a new invasion chamber. The same procedure was repeated three times to select for highly invasive cells designated KYSE410-I3 and KYSE510-I3 (Figure 1A, left panel).

### cDNA microarray and ingenuity pathway analysis (IPA)

Human Genome U133 Plus 2.0 GeneChip (Affymetrix Inc., Santa Clara, CA, USA) was used for genome-wide mRNA expression profiling. Total RNA was isolated using TRIZOL Reagent (Invitrogen), and the quality of total RNA was assessed with the Agilent 2100 Bioanalyzer Platform. Double-stranded cDNA was synthesized by reverse transcription from total RNA, and then *in vitro* transcription was performed to produce biotin-labeled cRNA from the cDNA. The cRNA is fragmented before hybridization. After washing and staining with the GeneChip Fluidics Station 450 (Affymetrix), the genechips were scanned with the GeneChip Scanner 3000 (Affymetrix). Scanned output files were analyzed with GeneSpring GX 12.0 software (Agilent Technologies, Inc., Santa Clara, CA, USA). A fold difference of > 2.0 was defined as differentially expressed. IPA software (Ingenuity Systems, Redwood City, CA, USA) was used for pathway analysis. The Gene ID of the differentially expressed genes in KYSE410-I3 cells and the corresponding fold-change were uploaded into IPA software (Ingenuity Systems, Redwood City, CA, USA). The Core Analysis tool, Gene Ontology analysis, and the Fisher's Exact Test in IPA were used to identify statistically significant associations between differentially expressed genes and cellular/molecular pathways. We configured the core analysis to report Benjamini–Hochberg corrected *P* values.

### Transfection and establishment of stable cell lines

The plasmid expressing phosphatase and tensin homolog (*PTEN*), i.e. pcDNA3-PTEN [48], and the vector control pcDNA3-GFP [49] were gifts from William Sellers (Dana Faber Cancer Institute, Boston, MA, USA) and Alonzo Ross (University of Massachusetts, Worcester, MA, USA), respectively (Addgene plasmids 10759 and 20738; Addgene, Cambridge, MA, USA). Transfection and establishment of stable cell lines were performed as described previously [7].

### Western blot

Details on preparation of cell and tumor lysates, and Western blotting were described previously [50]. The primary antibodies used included the following purchased from Cell Signaling Technology (Beverly, MA, USA): p-AKT (Ser473) and AKT for detecting the phosphorylated form and total AKT respectively, PTEN for confirming successful ectopic expression of PTEN after ESCC cells were transfected with PTEN-expressing plasmid, Src and p-Src (Tyr416) for detecting total Src protein and its phosphorylation status in order to evaluate activation of Src pathway. Primary antibodies against E-cadherin (BD Biosciences) and N-cadherin (Santa Cruz Biotechnology, Santa Cruz, CA, USA) were used to study the role of PI3K/AKT in EMT. Antibody against human-specific cytokeratin-8 (Epitomics, Burlingame, CA, USA) was used to detect presence of human cancer cells in the lungs of experimental metastasis model. Actin, serving as loading control, was detected using an antibody from Santa Cruz Biotechnology.

### Quantitative real-time PCR

The qRT-PCR was performed as described previously [51]. In brief, total RNA was isolated using Trizol reagent according to the manufacturer's protocol (Invitrogen). The cDNA was synthesized using the PrimeScript™ II 1st Strand cDNA Synthesis Kit (Takara, Dalian, China). The mRNA expression levels of *ANKRD1*, *TTC3* and of *GAPDH* as internal control, were detected by real-time PCR using SYBR® Premix Ex Taq™ II (Takara). The primers used were: 5′-ACGCCAAAGACAGAGAAGGA-3′ (forward) and 5′- CCAGTGTAGCACCAGATCCA-3′ (reverse) for *ANKRD1*; 5′-AGCACTGAGCTTGCT GGTTT-3′ (forward) and 5′-CTTGTCCTTTCCTGGG TTTG-3′ (reverse) for *TTC3*; 5′-AAGGTGAAGGTC GGAGTCAA-3′ (forward) and 5′-GACAAGCTTCCC GTTCTCAG-3′ (reverse) for *GAPDH*.

### 3-(4,5-Dimethyl thiazol-2-yl)-2,5-diphenyl tetrazolium bromide (MTT) assay

MTT assay was performed to measure cell viability as described before [52].

### *In vitro* cell migration and invasion assays

*In vitro* cell migration assays were carried out with the use of uncoated Transwell chambers (Corning, New York, NY) [52]. *In vitro* cell invasion assays were performed with the use of BD BioCoat Matrigel Invasion chambers (BD Biosciences) according to manufacturer's instructions [7].

### Experimental metastasis assay in nude mice

Female BALB/c nude mice aged 6-8 weeks were maintained under standard conditions and cared for according to the institutional guidelines for animal care. About 1 × 10^6^ KYSE150 cells with ectopic luciferase expression (i.e. KYSE150-Luc) were suspended in phosphate buffered saline and injected intravenously through the lateral tail vein of the mice [53]. Seven days after injection of cancer cells, the animals were treated twice weekly with PI3K inhibitor wortmannin (0.3 mg/kg or 0.6 mg/kg) or vehicle through intraperitoneal injection. The treatment regimen was designed based on published studies [54, 55] to minimize toxic effects on the animals. Metastatic activity was assessed by bioluminescent imaging with an IVIS Imaging System (Xenogen, Alameda, CA) after intraperitoneal injection of D-luciferin (Gold Biotechnology, St Louis, MO). The animals were euthanized at the end of the experiment, and the lungs were collected for further analyses including Western blotting and histological analysis. All the animal experiments were approved by the Committee on the Use of Live Animals in Teaching and Research of the University of Hong Kong.

### Tissue samples, tissue microarray and immunohistochemistry

Immunohistochemical staining was performed on paraffin sections of 28 cases of primary ESCCs and 15 cases of lymph nodes with metastatic ESCC in order to compare p-AKT expression pattern in cancer cells within primary tumors against that of metastasized ESCC cells. A tissue microarray (TMA) containing a separate cohort of 82 cases of primary ESCC (Supplementary Table 3) was also included to determine whether p-AKT expression is correlated with demographic features of patients and clinicopathological parameters. These specimens were collected from patients who underwent surgical resection of primary esophageal tumor at Queen Mary Hospital in Hong Kong, or the First Affiliated Hospital, Zhengzhou University in Zhengzhou, China, from 1989 to 2004. All clinical samples were obtained with informed consent of the patients. Ethical approval was also obtained from the Griffith University Human Research Ethics Committee (GU Ref Nos: MED/19/08/HREC and MSC/17/10/HREC) for retrospective use of ESCC tissues. The TMA was constructed using a Galileo CK3500 tissue Microarrayer (Integrated System Engineering Srl, Milano, Italy). Briefly, all representative donor blocks of ESCC were cut for haematoxylin & eosin staining to define the site of the representative regions of ESCC. From those regions, 3 cylindrical core tissue specimens (diameter = 0.6 mm) were acquired from each ESCC and arrayed into a new recipient paraffin block (35 × 20mm^2^). Paraffin-embedded sections and TMAs were deparaffinized in toluene and rehydrated in a graded series of ethanol solutions. Following antigen retrieval in citrate buffer (pH 6.0) and blocking with normal serum, the slides were incubated overnight with phospho-AKT (Ser473) antibody (Cell Signaling Technology), washed with phosphate buffered saline, and then incubated with biotinylated secondary antibody for 30 min at room temperature. Peroxidase-conjugated avidin-biotin complex was added to the sections and 3,3′-diaminobenzidine (Dako) was used as chromogen to visualize the immunostaining, followed by counterstaining with hematoxylin. The immunohistochemical staining for p-AKT nuclear expression was classified into four categories: no or negligible staining (score 0), weakly positive with less than 10% tumor cells stained (score 1+), moderate intensity with 10 to 50% cells stained (score 2+), and strong staining intensity in > 50% cells (score 3+). Cases with scores of 0 or 1+ were grouped as “low” expression, whereas those with a score of 2+ or 3+ were regarded as having high expression.

### Statistical analysis

The data were expressed as the mean ± SD and compared using ANOVA. All *in vitro* experiments were repeated at least three times. For the immunohistochemistry results, the data were entered into a computer database and the statistical analysis was performed using SPSS for Windows (version 22.0, IBM SPSS Inc., New York, NY). Fisher's exact test or Pearson Chi-Square was used for analyzing the association between expression level p-AKT and categorical clinicopathological variables. *P* values < 0.05 were deemed significant.

## SUPPLEMENTARY MATERIALS FIGURES AND TABLES






