# Supplementary material for: Significance of PI3K/AKT signaling pathway in metastasis of esophageal squamous cell carcinoma and its potential as a target for anti-metastasis therapy
Source: Oncotarget. 2017 Mar 17;8(24):38755–66. doi: 10.18632/oncotarget.16333 (PMC5503569; doi:10.18632/oncotarget.16333)
Supplement: Supplementary file 1 [file oncotarget-08-38755-s001.pdf]

Significance of PI3K/AKT signaling pathway in metastasis of esophageal squamous cell carcinoma and its potential as a target for anti-metastasis therapy

SUPPLEMENTARY MATERIALS

Supplementary Table 1: Differentially expressed genes (fold change < 2.0) between KYSE410-I3 and KYSE410 cells

See Supplementary File: 1

Supplementary Table 2: Correlation between p-AKT expression and clinicopathological variables in 82 cases of ESCC

	p-AKT			
**Parameters**	**Low**	**High**	**Total**	***P* value**
**Number**	47	35	82	-
**Age (year)**				
< 63	21	19	40	
≥ 63	23	19	42	1.000
**Size (cm)**				
< 5.0	20	14	34	
≥ 5.0	24	24	48	0.503
**Gender**				
Male	37	33	70	
Female	7	5	12	0.765
**Differentiation**				
Well and moderate	37	32	69	
Poor	7	6	13	1.0
**Location**				
Upper	5	1	6	
Middle or lower	39	37	76	0.209
**T-Stage**				
1	2	0	2	
2	0	2	2	
3	30	28	58	
4	12	8	20	0.217
**N-Stage**				
N0	13	10	23	
N1	31	28	59	0.809
**M-Stage**				
M0	43	37	80	
M1	1	1	2	1.000

Abbreviations: T, tumor; N, nodal; M, metastasis.

Supplementary Table 3: Clinicopathological characteristics and p-AKT staining score of 82 cases of ESCC in TMA

See Supplementary File: 2
