# Supplementary material for: Significance of PI3K/AKT signaling pathway in metastasis of esophageal squamous cell carcinoma and its potential as a target for anti-metastasis therapy
Source: Oncotarget. 2017 Mar 17;8(24):38755–66. doi: 10.18632/oncotarget.16333 (PMC5503569; doi:10.18632/oncotarget.16333)
Supplement: Supplementary file 2 [file oncotarget-08-38755-s002.docx]

**Supplementary Table S1.** Differentially expressed genes (fold change > 2.0) between KYSE410-I3 and KYSE410 cells.

| **ID** | **Symbol** | **Entrez Gene Name** | **Fold Change** |
| --- | --- | --- | --- |
| 201858_s_at | SRGN | serglycin | 5.643 |
| 1557779_at | LINC01468 | long intergenic non-protein coding RNA 1468 | 5.357 |
| 234994_at | TMEM200A | transmembrane protein 200A | 5.245 |
| 202016_at | MEST | mesoderm specific transcript | 4.967 |
| 1559584_a_at | C16orf54 | chromosome 16 open reading frame 54 | 4.776 |
| 203634_s_at | CPT1A | carnitine palmitoyltransferase 1A | 4.759 |
| 206157_at | PTX3 | pentraxin 3 | 4.44 |
| 204614_at | SERPINB2 | serpin family B member 2 | 4.334 |
| 209278_s_at | TFPI2 | tissue factor pathway inhibitor 2 | 4.295 |
| 226926_at | DMKN | dermokine | 4.182 |
| 39402_at | IL1B | interleukin 1 beta | 3.841 |
| 226847_at | FST | follistatin | 3.829 |
| 228144_at | ZNF300 | zinc finger protein 300 | 3.782 |
| 203887_s_at | THBD | thrombomodulin | 3.608 |
| 201996_s_at | SPEN | spen family transcriptional repressor | 3.302 |
| 213415_at | CLIC2 | chloride intracellular channel 2 | 3.069 |
| 204236_at | FLI1 | Fli-1 proto-oncogene, ETS transcription factor | 3.045 |
| 1553605_a_at | ABCA13 | ATP binding cassette subfamily A member 13 | 3.028 |
| 204363_at | F3 | coagulation factor III, tissue factor | 3.024 |
| 208891_at | DUSP6 | dual specificity phosphatase 6 | 2.997 |
| 209101_at | CTGF | connective tissue growth factor | 2.781 |
| 219179_at | DACT1 | dishevelled binding antagonist of beta catenin 1 | 2.767 |
| 204396_s_at | GRK5 | G protein-coupled receptor kinase 5 | 2.719 |
| 223704_s_at | DMRT2 | doublesex and mab-3 related transcription factor 2 | 2.629 |
| 226218_at | IL7R | interleukin 7 receptor | 2.586 |
| 218656_s_at | LHFP | lipoma HMGIC fusion partner | 2.571 |
| 1558686_at | MPV17L | MPV17 mitochondrial inner membrane protein like | 2.532 |
| 216268_s_at | JAG1 | jagged 1 | 2.528 |
| 212298_at | NRP1 | neuropilin 1 | 2.493 |
| 235236_at | FAM196B | family with sequence similarity 196 member B | 2.49 |
| 210118_s_at | IL1A | interleukin 1 alpha | 2.452 |
| 226368_at | CHST11 | carbohydrate sulfotransferase 11 | 2.443 |
| 209765_at | ADAM19 | ADAM metallopeptidase domain 19 | 2.426 |
| 213338_at | TMEM158 | transmembrane protein 158 (gene/pseudogene) | 2.422 |
| 201721_s_at | LAPTM5 | lysosomal protein transmembrane 5 | 2.412 |
| 206290_s_at | RGS7 | regulator of G-protein signaling 7 | 2.382 |
| 232054_at | PCDH20 | protocadherin 20 | 2.361 |
| 213652_at | PCSK5 | proprotein convertase subtilisin/kexin type 5 | 2.333 |
| 202998_s_at | LOXL2 | lysyl oxidase like 2 | 2.307 |
| 224724_at | SULF2 | sulfatase 2 | 2.303 |
| 202409_at | IGF2 | insulin like growth factor 2 | 2.294 |
| 202686_s_at | AXL | AXL receptor tyrosine kinase | 2.243 |
| 205289_at | BMP2 | bone morphogenetic protein 2 | 2.232 |
| 218723_s_at | RGCC | regulator of cell cycle | 2.228 |
| 1552487_a_at | BNC1 | basonuclin 1 | 2.222 |
| 205542_at | STEAP1 | STEAP family member 1 | 2.2 |
| 206029_at | ANKRD1 | ankyrin repeat domain 1 | 2.199 |
| 205532_s_at | CDH6 | cadherin 6 | 2.176 |
| 201466_s_at | JUN | Jun proto-oncogene, AP-1 transcription factor subunit | 2.145 |
| 1552546_a_at | LETM2 | leucine zipper and EF-hand containing transmembrane protein 2 | 2.133 |
| 202664_at | WIPF1 | WAS/WASL interacting protein family member 1 | 2.107 |
| 224461_s_at | AIFM2 | apoptosis inducing factor, mitochondria associated 2 | 2.103 |
| 232406_at | LOC105372526 | uncharacterized LOC105372526 | 2.089 |
| 242005_at | LINC00973 | long intergenic non-protein coding RNA 973 | 2.077 |
| 219093_at | PID1 | phosphotyrosine interaction domain containing 1 | 2.068 |
| 201645_at | TNC | tenascin C | 2.058 |
| 206237_s_at | NRG1 | neuregulin 1 | 2.05 |
| 209708_at | MOXD1 | monooxygenase DBH like 1 | 2.047 |
| 220169_at | TMEM156 | transmembrane protein 156 | 2.03 |
| 204115_at | GNG11 | G protein subunit gamma 11 | 2.014 |
| 224480_s_at | GPAT3 | glycerol-3-phosphate acyltransferase 3 | 2.013 |
| 205969_at | AADAC | arylacetamide deacetylase | 2.008 |
| 221435_x_at | HYI | hydroxypyruvate isomerase (putative) | 2.006 |
| 207222_at | PLA2G10 | phospholipase A2 group X | -2.001 |
| 203639_s_at | FGFR2 | fibroblast growth factor receptor 2 | -2.002 |
| 212240_s_at | PIK3R1 | phosphoinositide-3-kinase regulatory subunit 1 | -2.011 |
| 223655_at | CD163L1 | CD163 molecule like 1 | -2.013 |
| 204199_at | RALGPS1 | Ral GEF with PH domain and SH3 binding motif 1 | -2.013 |
| 239486_at | REL | REL proto-oncogene, NF-kB subunit | -2.014 |
| 210073_at | ST8SIA1 | ST8 alpha-N-acetyl-neuraminide alpha-2,8-sialyltransferase 1 | -2.016 |
| 222912_at | ARRB1 | arrestin beta 1 | -2.017 |
| 207949_s_at | ICA1 | islet cell autoantigen 1 | -2.018 |
| 221511_x_at | CCPG1 | cell cycle progression 1 | -2.02 |
| 203562_at | FEZ1 | fasciculation and elongation protein zeta 1 | -2.023 |
| 222830_at | GRHL1 | grainyhead like transcription factor 1 | -2.024 |
| 203021_at | SLPI | secretory leukocyte peptidase inhibitor | -2.026 |
| 235343_at | VASH2 | vasohibin 2 | -2.027 |
| 225601_at | HMGB3 | high mobility group box 3 | -2.03 |
| 214079_at | DHRS2 | dehydrogenase/reductase 2 | -2.031 |
| 210665_at | TFPI | tissue factor pathway inhibitor | -2.034 |
| 239278_at | BCAS4 | breast carcinoma amplified sequence 4 | -2.035 |
| 205487_s_at | VGLL1 | vestigial like family member 1 | -2.035 |
| 235110_at | PLA2G16 | phospholipase A2 group XVI | -2.038 |
| 231424_at | SLC5A12 | solute carrier family 5 member 12 | -2.038 |
| 226164_x_at | RIMKLB | ribosomal modification protein rimK like family member B | -2.04 |
| 221305_s_at | UGT1A7 (includes others) | UDP glucuronosyltransferase family 1 member A10 | -2.043 |
| 202784_s_at | NNT | nicotinamide nucleotide transhydrogenase | -2.048 |
| 223125_s_at | C1orf21 | chromosome 1 open reading frame 21 | -2.052 |
| 209301_at | CA2 | carbonic anhydrase 2 | -2.054 |
| 220133_at | ODAM | odontogenic, ameloblast asssociated | -2.054 |
| 201649_at | UBE2L6 | ubiquitin conjugating enzyme E2 L6 | -2.058 |
| 210656_at | EED | embryonic ectoderm development | -2.06 |
| 1554679_a_at | LAPTM4B | lysosomal protein transmembrane 4 beta | -2.06 |
| 240979_at | YWHAEP7 | tyrosine 3-monooxygenase/tryptophan 5-monooxygenase activation protein epsilon pseudogene 7 | -2.06 |
| 218983_at | C1RL | complement C1r subcomponent like | -2.062 |
| 212657_s_at | IL1RN | interleukin 1 receptor antagonist | -2.063 |
| 220484_at | MCOLN3 | mucolipin 3 | -2.063 |
| 204664_at | ALPP | alkaline phosphatase, placental | -2.064 |
| 223748_at | SLC4A11 | solute carrier family 4 member 11 | -2.064 |
| 231944_at | ERO1B | endoplasmic reticulum oxidoreductase 1 beta | -2.065 |
| 219771_at | TBC1D8B | TBC1 domain family member 8B | -2.067 |
| 210148_at | HIPK3 | homeodomain interacting protein kinase 3 | -2.073 |
| 1569472_s_at | TTC3 | tetratricopeptide repeat domain 3 | -2.077 |
| 223183_at | AGPAT3 | 1-acylglycerol-3-phosphate O-acyltransferase 3 | -2.082 |
| 213916_at | ZNF20 | zinc finger protein 20 | -2.084 |
| 225666_at | TMTC4 | transmembrane and tetratricopeptide repeat containing 4 | -2.085 |
| 207417_s_at | ZNF177 | zinc finger protein 177 | -2.085 |
| 1553971_a_at | GATS | GATS, stromal antigen 3 opposite strand | -2.087 |
| 1554588_a_at | TTC30B | tetratricopeptide repeat domain 30B | -2.088 |
| 217437_s_at | TACC1 | transforming acidic coiled-coil containing protein 1 | -2.093 |
| 233068_at | CRIPT | CXXC repeat containing interactor of PDZ3 domain | -2.105 |
| 215509_s_at | BUB1 | BUB1 mitotic checkpoint serine/threonine kinase | -2.106 |
| 228058_at | ZG16B | zymogen granule protein 16B | -2.108 |
| 205442_at | MFAP3L | microfibrillar associated protein 3 like | -2.111 |
| 205535_s_at | PCDH7 | protocadherin 7 | -2.125 |
| 1554397_s_at | UEVLD | UEV and lactate/malate dehyrogenase domains | -2.129 |
| 224046_s_at | PDE7A | phosphodiesterase 7A | -2.142 |
| 218692_at | SYBU | syntabulin | -2.143 |
| 219682_s_at | TBX3 | T-box 3 | -2.143 |
| 239764_at | ITPR1-AS1 | ITPR1 antisense RNA 1 (head to head) | -2.15 |
| 201369_s_at | ZFP36L2 | ZFP36 ring finger protein like 2 | -2.161 |
| 238681_at | GDPD1 | glycerophosphodiester phosphodiesterase domain containing 1 | -2.164 |
| 211596_s_at | LRIG1 | leucine rich repeats and immunoglobulin like domains 1 | -2.167 |
| 233520_s_at | CMYA5 | cardiomyopathy associated 5 | -2.169 |
| 219310_at | SYNDIG1 | synapse differentiation inducing 1 | -2.176 |
| 231738_at | PCDHB7 | protocadherin beta 7 | -2.183 |
| 206515_at | CYP4F3 | cytochrome P450 family 4 subfamily F member 3 | -2.187 |
| 221029_s_at | WNT5B | Wnt family member 5B | -2.188 |
| 206433_s_at | SPOCK3 | SPARC/osteonectin, cwcv and kazal like domains proteoglycan 3 | -2.19 |
| 207847_s_at | MUC1 | mucin 1, cell surface associated | -2.191 |
| 236193_at | HIST1H2BC | histone cluster 1 H2B family member c | -2.204 |
| 207308_at | SLCO1A2 | solute carrier organic anion transporter family member 1A2 | -2.21 |
| 207076_s_at | ASS1 | argininosuccinate synthase 1 | -2.212 |
| 205003_at | DOCK4 | dedicator of cytokinesis 4 | -2.216 |
| 1553694_a_at | PIK3C2A | phosphatidylinositol-4-phosphate 3-kinase catalytic subunit type 2 alpha | -2.223 |
| 238781_at | SREK1 | splicing regulatory glutamic acid and lysine rich protein 1 | -2.231 |
| 219181_at | LIPG | lipase G, endothelial type | -2.233 |
| 230951_at | EPB41L5 | erythrocyte membrane protein band 4.1 like 5 | -2.238 |
| 203854_at | CFI | complement factor I | -2.242 |
| 215236_s_at | PICALM | phosphatidylinositol binding clathrin assembly protein | -2.245 |
| 204378_at | BCAS1 | breast carcinoma amplified sequence 1 | -2.253 |
| 203066_at | CHST15 | carbohydrate sulfotransferase 15 | -2.254 |
| 208146_s_at | CPVL | carboxypeptidase, vitellogenic like | -2.254 |
| 213568_at | OSR2 | odd-skipped related transciption factor 2 | -2.254 |
| 218901_at | PLSCR4 | phospholipid scramblase 4 | -2.261 |
| 232843_s_at | DOCK8 | dedicator of cytokinesis 8 | -2.281 |
| 232654_s_at | UGT1A6 | UDP glucuronosyltransferase family 1 member A6 | -2.281 |
| 217865_at | RNF130 | ring finger protein 130 | -2.285 |
| 225957_at | CREBRF | CREB3 regulatory factor | -2.29 |
| 222062_at | IL27RA | interleukin 27 receptor subunit alpha | -2.295 |
| 211479_s_at | HTR2C | 5-hydroxytryptamine receptor 2C | -2.308 |
| 1556267_at | MYRFL | myelin regulatory factor-like | -2.317 |
| 225095_at | SPTLC2 | serine palmitoyltransferase long chain base subunit 2 | -2.325 |
| 1569157_s_at | ZNF846 | zinc finger protein 846 | -2.327 |
| 39248_at | AQP3 | aquaporin 3 (Gill blood group) | -2.353 |
| 213240_s_at | KRT4 | keratin 4 | -2.359 |
| 210503_at | MAGEA11 | MAGE family member A11 | -2.37 |
| 226022_at | SASH1 | SAM and SH3 domain containing 1 | -2.384 |
| 211559_s_at | CCNG2 | cyclin G2 | -2.395 |
| 226424_at | CAPS | calcyphosine | -2.416 |
| 204749_at | NAP1L3 | nucleosome assembly protein 1 like 3 | -2.421 |
| 230081_at | PLCXD3 | phosphatidylinositol specific phospholipase C X domain containing 3 | -2.425 |
| 218454_at | PLBD1 | phospholipase B domain containing 1 | -2.441 |
| 212841_s_at | PPFIBP2 | PPFIA binding protein 2 | -2.46 |
| 226622_at | MUC20 | mucin 20, cell surface associated | -2.462 |
| 203263_s_at | ARHGEF9 | Cdc42 guanine nucleotide exchange factor 9 | -2.483 |
| 226875_at | DOCK11 | dedicator of cytokinesis 11 | -2.484 |
| 202730_s_at | PDCD4 | programmed cell death 4 (neoplastic transformation inhibitor) | -2.494 |
| 203217_s_at | ST3GAL5 | ST3 beta-galactoside alpha-2,3-sialyltransferase 5 | -2.495 |
| 230964_at | FREM2 | FRAS1 related extracellular matrix protein 2 | -2.504 |
| 203710_at | ITPR1 | inositol 1,4,5-trisphosphate receptor type 1 | -2.514 |
| 215071_s_at | HIST1H2AC | histone cluster 1 H2A family member c | -2.594 |
| 35974_at | LRMP | lymphoid restricted membrane protein | -2.602 |
| 206525_at | GABRR1 | gamma-aminobutyric acid type A receptor rho1 subunit | -2.616 |
| 222351_at | PPP2R1B | protein phosphatase 2 scaffold subunit Abeta | -2.628 |
| 208083_s_at | ITGB6 | integrin subunit beta 6 | -2.666 |
| 241763_s_at | FBXO32 | F-box protein 32 | -2.67 |
| 205248_at | DOPEY2 | dopey family member 2 | -2.671 |
| 213792_s_at | INSR | insulin receptor | -2.674 |
| 203504_s_at | ABCA1 | ATP binding cassette subfamily A member 1 | -2.716 |
| 205342_s_at | SULT1C2 | sulfotransferase family 1C member 2 | -2.734 |
| 229720_at | BAG1 | BCL2 associated athanogene 1 | -2.74 |
| 227020_at | YPEL2 | yippee like 2 | -2.748 |
| 229385_s_at | TINCR | tissue differentiation-inducing non-protein coding RNA | -2.76 |
| 209755_at | NMNAT2 | nicotinamide nucleotide adenylyltransferase 2 | -2.776 |
| 211549_s_at | HPGD | hydroxyprostaglandin dehydrogenase 15-(NAD) | -2.799 |
| 221024_s_at | SLC2A10 | solute carrier family 2 member 10 | -2.802 |
| 202688_at | TNFSF10 | tumor necrosis factor superfamily member 10 | -2.808 |
| 203571_s_at | ADIRF | adipogenesis regulatory factor | -2.815 |
| 229497_at | ANKDD1A | ankyrin repeat and death domain containing 1A | -2.816 |
| 219195_at | PPARGC1A | PPARG coactivator 1 alpha | -2.846 |
| 204446_s_at | ALOX5 | arachidonate 5-lipoxygenase | -2.872 |
| 232983_s_at | SERGEF | secretion regulating guanine nucleotide exchange factor | -2.882 |
| 239889_at | SERP2 | stress associated endoplasmic reticulum protein family member 2 | -2.894 |
| 232099_at | PCDHB16 | protocadherin beta 16 | -2.921 |
| 204720_s_at | DNAJC6 | DnaJ heat shock protein family (Hsp40) member C6 | -2.923 |
| 225911_at | NPNT | nephronectin | -2.924 |
| 241365_at | SATB1 | SATB homeobox 1 | -2.924 |
| 205319_at | PSCA | prostate stem cell antigen | -2.964 |
| 218541_s_at | C8orf4 | chromosome 8 open reading frame 4 | -3.033 |
| 221523_s_at | RRAGD | Ras related GTP binding D | -3.037 |
| 205590_at | RASGRP1 | RAS guanyl releasing protein 1 | -3.064 |
| 204567_s_at | ABCG1 | ATP binding cassette subfamily G member 1 | -3.184 |
| 226158_at | KLHL24 | kelch like family member 24 | -3.317 |
| 201860_s_at | PLAT | plasminogen activator, tissue type | -3.317 |
| 213385_at | CHN2 | chimerin 2 | -3.323 |
| 218559_s_at | MAFB | MAF bZIP transcription factor B | -3.344 |
| 202748_at | GBP2 | guanylate binding protein 2 | -3.455 |
| 206201_s_at | MEOX2 | mesenchyme homeobox 2 | -3.472 |
| 242656_at | GTF2H1 | general transcription factor IIH subunit 1 | -3.488 |
| 214321_at | NOV | nephroblastoma overexpressed | -3.5 |
| 202291_s_at | MGP | matrix Gla protein | -3.507 |
| 203185_at | RASSF2 | Ras association domain family member 2 | -3.509 |
| 232276_at | HS6ST3 | heparan sulfate 6-O-sulfotransferase 3 | -3.51 |
| 212224_at | ALDH1A1 | aldehyde dehydrogenase 1 family member A1 | -3.57 |
| 210095_s_at | IGFBP3 | insulin like growth factor binding protein 3 | -3.596 |
| 207935_s_at | KRT13 | keratin 13 | -3.604 |
| 202948_at | IL1R1 | interleukin 1 receptor type 1 | -3.687 |
| 222853_at | FLRT3 | fibronectin leucine rich transmembrane protein 3 | -3.988 |
| 206584_at | LY96 | lymphocyte antigen 96 | -4.001 |
| 229649_at | NRXN3 | neurexin 3 | -4.142 |
| 228256_s_at | EPB41L4A | erythrocyte membrane protein band 4.1 like 4A | -4.147 |
| 213456_at | SOSTDC1 | sclerostin domain containing 1 | -4.177 |
| 228067_at | KIAA1211L | KIAA1211 like | -4.247 |
| 212531_at | LCN2 | lipocalin 2 | -4.391 |
| 220625_s_at | ELF5 | E74 like ETS transcription factor 5 | -4.4 |
| 205413_at | MPPED2 | metallophosphoesterase domain containing 2 | -4.598 |
| 201141_at | GPNMB | glycoprotein nmb | -4.628 |
| 227702_at | CYP4X1 | cytochrome P450 family 4 subfamily X member 1 | -4.958 |
| 209118_s_at | TUBA1A | tubulin alpha 1a | -6.002 |
| 201641_at | BST2 | bone marrow stromal cell antigen 2 | -7.996 |
| 229554_at | LUM | lumican | -8.351 |
