# Supplementary material for: Significance of PI3K/AKT signaling pathway in metastasis of esophageal squamous cell carcinoma and its potential as a target for anti-metastasis therapy
Source: Oncotarget. 2017 Mar 17;8(24):38755–66. doi: 10.18632/oncotarget.16333 (PMC5503569; doi:10.18632/oncotarget.16333)
Supplement: Supplementary file 3 [file oncotarget-08-38755-s003.pdf]

Supplementary Table S3. Clinicopathological characteristics and p-AKT staining score of 82 cases of ESCC in TMA.

Case No.	Age	Gender	Location	Size (cm)	Differentiation	T-stage	N-stage	M-stage	Stage	p-AKT score
1	68	M	M	3	M	3	1	0	3	3
2	64	F	M	4	P	3	0	0	2	1
3	74	M	M	3	M	3	1	0	3	1
4	59	M	M	9.5	W	4	1	0	3	2
5	61	M	M	5.5	W	3	1	0	3	1
6	69	M	M	7	M	3	1	0	3	3
7	61	F	M	4	P	3	1	0	3	3
8	66	F	M	7.5	M	3	1	0	3	3
9	59	F	M	11	M	4	1	0	3	2
10	61	F	U	7	M	4	1	0	3	1
11	57	M	M	1	W	3	1	0	3	3
12	67	F	L	3.5	M	3	1	0	3	1
13	74	F	L	4	W	3	1	0	3	1
14	57	M	M	7	M	3	1	0	3	1
15	54	M	L	6	M	3	1	0	3	1
16	58	M	L	5	M	3	1	0	3	3
17	57	M	M	5	M	3	1	0	3	3
18	54	M	U	4	M	4	1	0	3	1
19	69	M	L	7.5	W	4	0	0	3	1
20	65	M	L	9	W	4	0	0	3	1
21	56	M	L	4	W	3	1	0	3	1
22	50	M	L	5	W	3	1	0	3	3
23	54	M	M	4.5	P	3	1	0	3	1
24	61	M	L	3.2	M	3	1	0	3	1
25	54	M	M	8	W	3	0	0	2	1
26	75	M	U	5	W	3	1	0	3	1
27	73	M	L	5	M	3	1	0	3	1
28	63	M	M	4.5	W	3	1	0	3	1
29	51	M	M	4	P	3	1	0	3	1
30	57	M	M	10.5	P	4	1	0	3	2
31	62	M	M	5.5	W	4	1	0	3	1
32	63	M	M	10	W	4	1	0	3	1
33	49	M	L	2.5	M	3	1	1	4	2
34	66	M	L	6	W	3	1	0	3	3
35	48	M	L	4	W	3	0	0	1	3
36	66	M	L	5	M	4	1	0	3	3
37	65	M	L	7	P	3	1	0	3	2
38	66	M	M	6	W	3	0	0	2	2
39	60	F	M	4	M	4	1	0	3	1
40	55	M	L	7	M	3	1	0	3	1
41	66	M	L	3	P	3	1	0	3	1
42	58	M	M	3.5	W	1	0	0	1	1
43	41	M	L	9	M	3	1	0	3	1
44	39	M	M	12	M	4	1	0	3	2

Supplementary Table S3. (continued)

Case No.	Age	Gender	Location	Size (cm)	Differentiation	T stage	N stage	M stage	Stage	p-AKT staining
45	46	M	M	5	W	3	1	0	3	1
46	60	M	M	4	M	4	1	0	3	1
47	59	F	M	7.5	M	3	1	0	3	1
48	55	M	L	1	P	1	0	0	1	1
49	53	M	M	4.5	W	3	0	0	2	2
50	77	M	L	2.5	M	3	1	0	3	1
51	76	M	M	5	W	4	0	0	3	1
52	58	M	M	3.5	W	3	1	0	3	2
53	83	M	M	4	M	3	1	0	3	1
54	74	M	M	7	W	4	0	0	3	1
55	72	M	L	5	M	3	1	0	3	1
56	78	M	M	6.5	P	4	0	0	3	1
57	80	M	U	3	M	4	0	0	3	1
58	43	M	L	4	M	3	1	0	3	1
59	61	M	L	5	W	3	1	0	3	2
60	62	M	M	5	M	4	1	0	3	3
61	70	F	M	3.5	M	2	0	0	2	3
62	65	M	M	2.5	M	3	0	0	2	1
63	75	F	M	6	P	3	0	0	2	1
64	61	M	L	6.5	W	3	1	0	3	1
65	40	M	M	7.5	P	3	1	0	3	2
66	77	M	U	5	W	3	1	1	4	1
67	47	M	U	4	M	4	1	0	3	3
68	64	M	M	5	P	3	0	0	2	3
69	68	M	M	4.5	M	3	1	0	3	3
70	74	M	M	6.5	W	3	1	0	3	2
71	67	M	M	4.5	W	3	0	0	2	2
72	66	M	L	3.5	M	3	1	0	3	3
73	63	M	L	4	M	3	0	0	2	2
74	64	M	L	6	M	3	0	0	2	1
75	72	M	M	3	P	3	1	0	3	2
76	66	M	L	7	W	3	1	0	3	2
77	60	M	M	6.5	W	4	1	0	3	3
78	67	M	L	5	W	3	0	0	2	3
79	73	M	M	5	M	3	1	0	3	1
80	46	F	M	5.5	M	2	0	0	2	3
81	71	M	M	6.5	W	3	0	0	2	2
82	65	M	L	7	M	3	1	0	3	2

Gender (M = male; F = female); U = upper oesophagus; M = middle oesophagus; L = lower oesophagus; W = well differentiated; M= moderately differentiated; P = poorly differentiated.
